# Self-reported benefits and risks of open water swimming to health, wellbeing and the environment: Cross-sectional evidence from a survey of Scottish swimmers

**DOI:** 10.1371/journal.pone.0290834

**Published:** 2023-08-28

**Authors:** David M. Oliver, Craig W. McDougall, Tony Robertson, Blair Grant, Nick Hanley, Richard S. Quilliam

**Affiliations:** 1 Biological and Environmental Sciences, Faculty of Natural Sciences, University of Stirling, Stirling, United Kingdom; 2 Scottish Collaboration for Public Health Research and Policy, School of Health in Social Science, University of Edinburgh, Edinburgh, United Kingdom; 3 European Centre for Environment and Human Health, University of Exeter Medical School, Truro, United Kingdom; 4 School of Biodiversity, One Health & Veterinary Medicine, University of Glasgow, Glasgow, United Kingdom; CIFRI: Central Inland Fisheries Research Institute, INDIA

## Abstract

Engaging with natural environments benefits human health by providing opportunities for social interactions, enhancing mental wellbeing and enabling outdoor spaces for physical exercise. Open water swimming has seen a rapid increase in popularity, partly due to the physical health benefits it can provide but also with the growing interest in (re)connecting with nature for environment-health interactions. Using a national-scale online survey of 717 open water swimmers, the aim of this study was to investigate patterns and trends in the perceived benefits and risks of open water swimming to both public health and the environment; and to understand whether these perceived risks and benefits vary across different typologies of swimmers and open water, or ‘blue space’, environments. Strong associations were found between the most important self-reported benefit associated with open water swimming and both participant age and the categorisation of their typical swim style. All but one of the age-groups surveyed perceived mental wellbeing benefits to be the most important benefit of open water swimming; whilst those aged over 65 identified physical rather than mental wellbeing benefits to be the most important outcome. Participants who preferred lake swimming reported greater concern regarding possible environmental damage caused by the increasing popularity of open water swimming compared to those engaging in river or sea swimming. However, the majority of participants perceived the risks to the environment from open water swimming to be minimal. Our study adds to the growing evidence that open water swimming is perceived by participants as benefitting their mental and physical wellbeing. Improved understanding of the benefits and risks of engaging with blue spaces used for open water swimming can contribute to co-designed policy development to promote safer, healthier and more sustainable outdoor recreation opportunities associated with this increasingly popular outdoor pursuit.

## Introduction

Engaging with natural environments benefits human health by providing opportunities for social interactions, enhancing mental wellbeing, and enabling outdoor spaces for physical exercise [1–3]. The public health benefits of exposure to green spaces, such as parks and woodlands, are well documented [4]. There is growing acknowledgement of the role of blue spaces (i.e., coastal environments and freshwater bodies) in delivering health and wellbeing benefits [5, 6]. The term ‘blue health’ recognises the psychological and stress recovery benefits that water can provide (beyond just the physical benefits of recreation in aquatic environments [7]). To understand the range of blue health opportunities available to the public there is a critical need to assess the role of diverse blue space environments such as coastal waters, lakes, wetlands, rivers and canals in promoting population health and wellbeing [8].

People engage with blue spaces in a variety of ways, including activities distant from a waterbody, such as waterside recreation or appreciating a seascape view; but also, through activities reliant on the water itself, such as surface water sports through to fully immersive experiences associated with open water, or wild swimming [5]. In common with health research on green spaces, studies on blue space have focused on the role of health and wellbeing benefits of proximity from, and visits to, environments of interest. However, blue spaces also offer an opportunity to understand unique health and wellbeing benefits arising from immersion in open water [9–11]. Despite this, little is known with respect to how perceived benefits and risks to health, wellbeing and the environment may vary according to preferred water environments for swimming (e.g., sea, lakes, and rivers) or across different typologies of swimmers (e.g., those striving to exercise versus those participating primarily to socialise) [12].

The increasing popularity of open water swimming has been linked to people’s interest in engaging with local environments and (re)connecting with nature to benefit from environment-health interactions [13], which coincides with a wider recognition of the health and wellbeing benefits of engaging with outdoor spaces more generally [14]. Lockdown restrictions in place during the COVID-19 pandemic may have also contributed to increased numbers of people choosing to explore local environments as indoor swimming venues were closed or restricted [15, 16]. However, increased outdoor recreation can exacerbate pressures on natural resources and landscapes, and result in environmental degradation if poorly managed [17]. Although open water swimming may not attract the same magnitude of visitors as might be expected at popular tourist hotspots, increased use of waterside areas at destination lakes as a direct consequence of the increased popularity of open water swimming may promote vegetation damage and erosion risks, and frustrate other local users [13].

Despite the physical health benefits of swimming being well recognised, medical improvements from outdoor swimming remain largely anecdotal [9]. Furthermore, while there is growing interest in the public health benefits of exposure to blue space and the variety of perceived positive health outcomes associated with immersive blue space experiences, there is also a need to understand the potential for increases in risk and trade-offs to public health. This may link to the increasing popularity of open water swimming and potential exposure to poor water quality [12]. For example, discharges of untreated wastewater from combined sewer overflows during wet weather events can have localised and downstream consequences in terms of risk to the environment, ecosystem service provision and human health [18], while warmer temperatures coupled with nutrient pollution increase the frequency of occurrence of harmful algal blooms [19].

The concept of waterscapes as therapeutic landscapes is gaining increased momentum and is attracting interdisciplinary research attention [20, 21], but many aquatic environments remain an under-utilised natural capital asset for nature-based health interventions, with unresolved issues regarding large-scale ‘blue-prescribing’ [22]. Developing a better understanding of how open water swimmers perceive the benefits and risks to their own health and wellbeing, and to the environment, from their nature-based activities can inform environmental decision-making associated with immersive blue space public health interventions. Further, it can contribute to co-designed policy development to promote safer, healthier and more sustainable outdoor recreation opportunities associated with this increasingly popular outdoor pursuit.

The aim of this study was to evaluate a national-scale dataset arising from an online survey of open-water swimmers in Scotland, and investigate patterns and trends in perceived benefits and risks of open water swimming to both public health and the environment. The specific objectives were to: (i) evaluate how perceived benefits and risks to health vary among different socio-demographic groups of the open water swimming community; (ii) assess whether preferred swimming environments, and different typologies of swims, influence the perceptions of health benefits and risks; and (iii) determine levels of concern regarding water quality and wider environmental degradation at swimming locations used by survey respondents.

## Materials and methods

### Data collection

A nationwide online survey was developed to obtain responses from the open-water swimming community in Scotland. This survey was designed using the JISC ‘Online Surveys’ software (https://www.jisc.ac.uk). The cross-sectional survey comprised 18 core questions regarding participation in open-water swimming, in addition to seeking socio-demographic information of each participant. The sample frame consisted of Scottish residents over the age of 18 who swim outdoors in Scottish open water environments (i.e., rivers, lochs/lakes and the sea). All participants volunteered to participate without a monetary incentive. All data were anonymous, with no identifying information available to the authors. Ethical approval was granted by the University of Stirling General University Ethics Panel (GUEP-2633).

A pilot version of the survey was tested on 10 participants to evaluate survey length and language prior to deployment, with only minimal edits subsequently made in order to improve clarity. A link to the main survey was then posted on a publicly accessible Facebook forum (‘Wild Swimming–Scotland’) with 97,000 members, enabling participant sign-up for eligible participants. The survey was live from 6^th^ July 2021 to 3^rd^ August 2021 and in total 717 participants were recruited and completed the survey.

The survey asked questions of the open water swimming community with respect to their experience and background, and their views on the risks and benefits to health and the environment from open water swimming. Participants were asked to identify their preferred outdoor water environment for swimming, the frequency and type of swimming they participate in, and how long they usually swam on a typical occasion. Participants were also asked to identify what they thought were the benefits of open water swimming and how they perceived particular risks to health and the environment from their open water swimming activities. A range of different question styles were used in the survey, including five-point Likert scale questions, multiple-choice questions, and open-ended questions. The Likert scale was used for its simplicity and ability to measure a series of attitude-related propositions and to use non-parametric tests such as Chi-Square (cross-tabulation) for statistical analyses [23]. Please refer to supporting information for a copy of the survey used (S1 File).

### Data analysis

Pearson’s Chi-Squared Test of Association was used to analyse the association between participants’ responses to different questions. Chi-square significance thresholds were set at P <0.05. Following on from Chi-square tests, Cramer’s Φ analysis (Φ_C_) was performed for any statistically significant associations to determine association strength. Thresholds of Φ_c_ for association strength were classified as: Very Strong >0.25, Strong >0.15, Moderate >0.10, Weak >0.05, Very weak >0 [24]. Minitab 18.0 software, Minitab Inc.; State College, PA, USA was used to determine the association between responses. For the open-ended / free-text questions, thematic analysis using a coding approach enabled the identification of key themes of interest.

## Results

In total, 717 completed survey responses were received (see data availability statement). The sample was heavily skewed by female participation (92%), although a range of age-groups and socio-economic backgrounds were recorded among participants (Table 1). The city of Edinburgh, Scotland’s capital and second largest urban conurbation, accounted for the largest proportion of survey participants (13%); however, there was good spatial coverage across Scotland, with surveys completed by participants from 30 of the 32 regions of the country. With respect to the average distance travelled to participate in open water swimming, 29% of participants travelled up to 1 km and just over half (53%) typically travelled up to 5 km to reach a waterbody. Overall, 86% of participants travelled within 20 km to participate in their open water swimming activity, with only 14% stating that, on average, they travelled over 20 km. Most of those surveyed stated that lochs and lakes were their preferred water environment for wild swimming (50%), with 44% and 6% indicating the sea and rivers, respectively.

**Table 1:** Socio-demographic summary of survey participants.

	Number and percentage of respondents	
	N	%
Total number of respondents	717	100
Gender		
Male	58	8.1
Female	657	91.6
Prefer not to say	2	0.3
Age		
18–24	25	3.5
25–34	88	12.3
35–44	164	22.9
45–54	279	38.9
55–64	141	19.7
65+	20	2.7
Annual household income		
**Less than £20k**	83	11.6
£20–29,999	96	13.4
£30–39,999	94	13.1
£40–49,999	91	12.7
£50–59,999	74	10.3
£60–69,999	54	7.5
Over £70k	150	20.9
Prefer not to say	75	10.5

**Fig 1:**
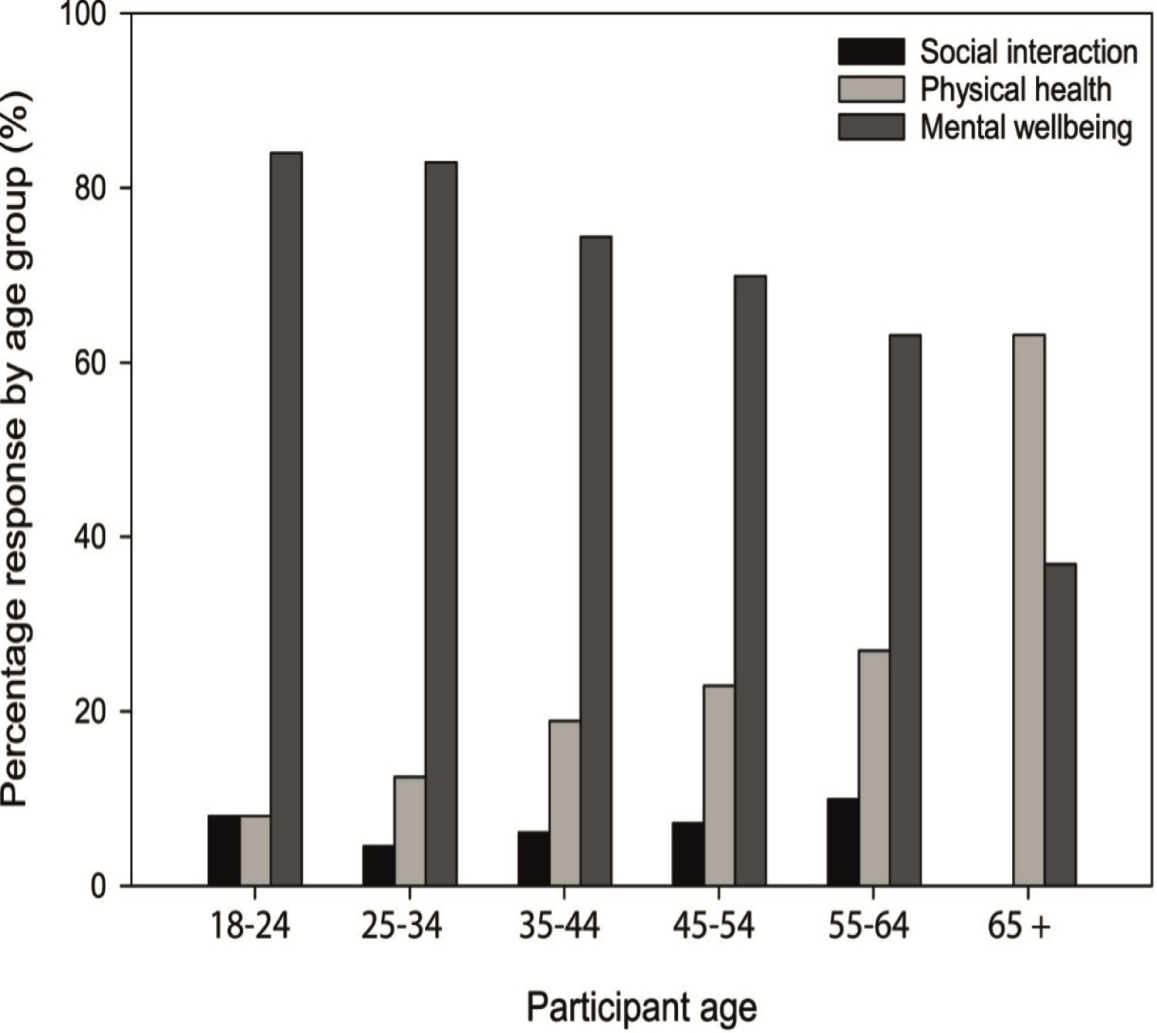
Perceptions of the most important benefit gained from open water swimming across different age categories.

**Fig 2:**
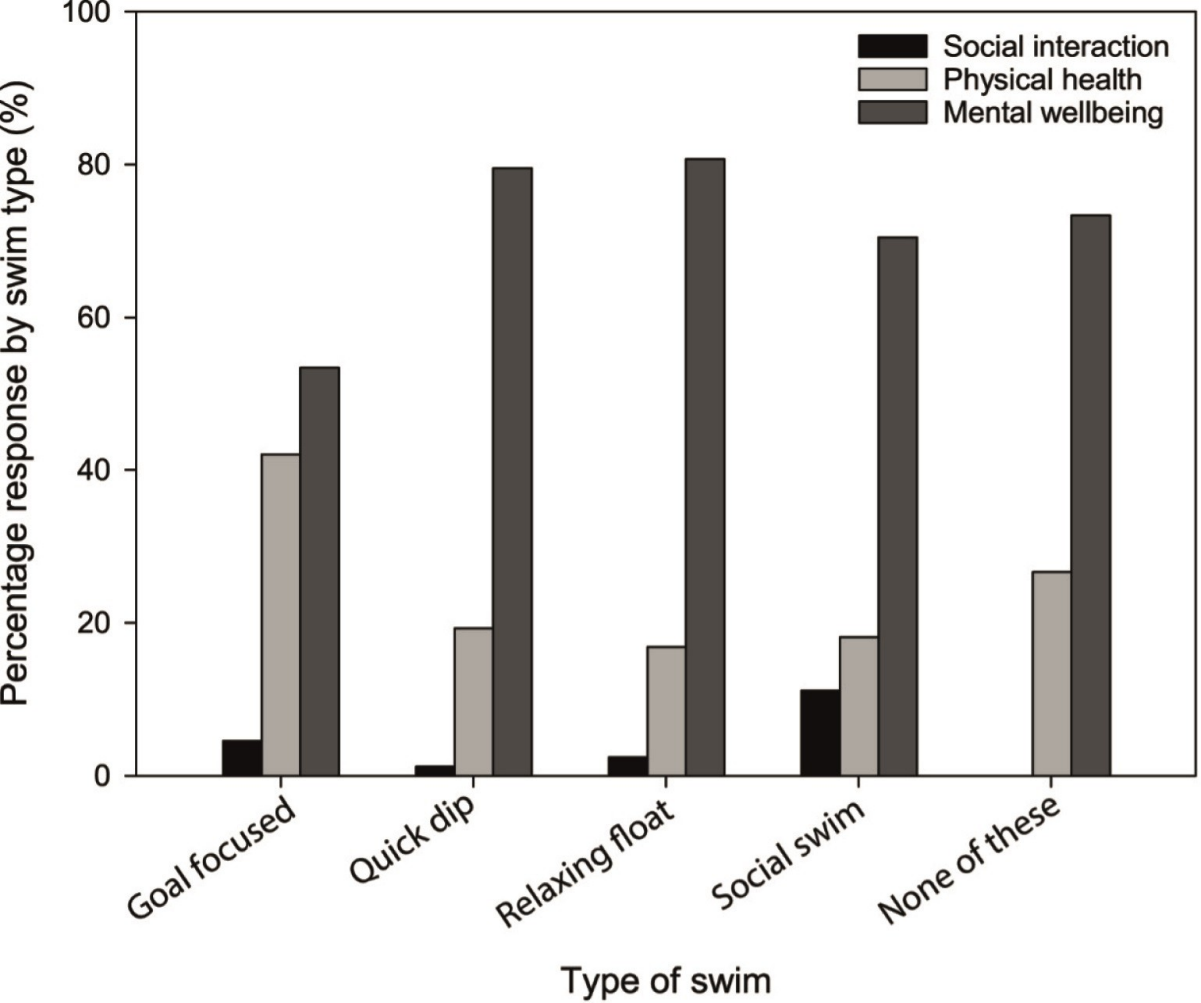
Perceptions of the most important benefit gained from open water swimming according to typical swim style.

Selecting from a choice of social interactions, physical health benefits and mental wellbeing benefits, there was a significant and strong association (P < 0.001; Φ_C_ = 0.16) between participant age and their most important self-reported benefit associated with open water swimming. All but one of the age-groups perceived mental wellbeing benefits to be the most important benefit of open water swimming; in contrast, those aged over 65 identified benefits to physical wellbeing as being more important than mental wellbeing (Fig 1). Likewise, a significant, strong association between the most important self-reported benefit associated with open water swimming and of their typical swim style (e.g., goal-focused, quick dip, relaxing float, social swim) was identified (P < 0.001; Φ_c_ = 0.18). Mental wellbeing benefits ranked highest across all swim styles (Fig 2); however, the proportion of participants rating mental wellbeing as the most important benefit was lowest among those participating in goal-focused swims (i.e., swims linked to training targets) (53% relative to > 70% for all other swim classifications). By contrast, for goal-focused swims, the proportion of participants recognising physical benefits to be the most important outcome was much higher than for other swim categories (42%). There was no association between the respondent’s most important perceived benefit and a range of other categorical factors such as annual income, distance travelled to go open water swimming, time spent in the water during a typical swim, and frequency of participating in open water swimming (P > 0.05).

**Fig 3:**
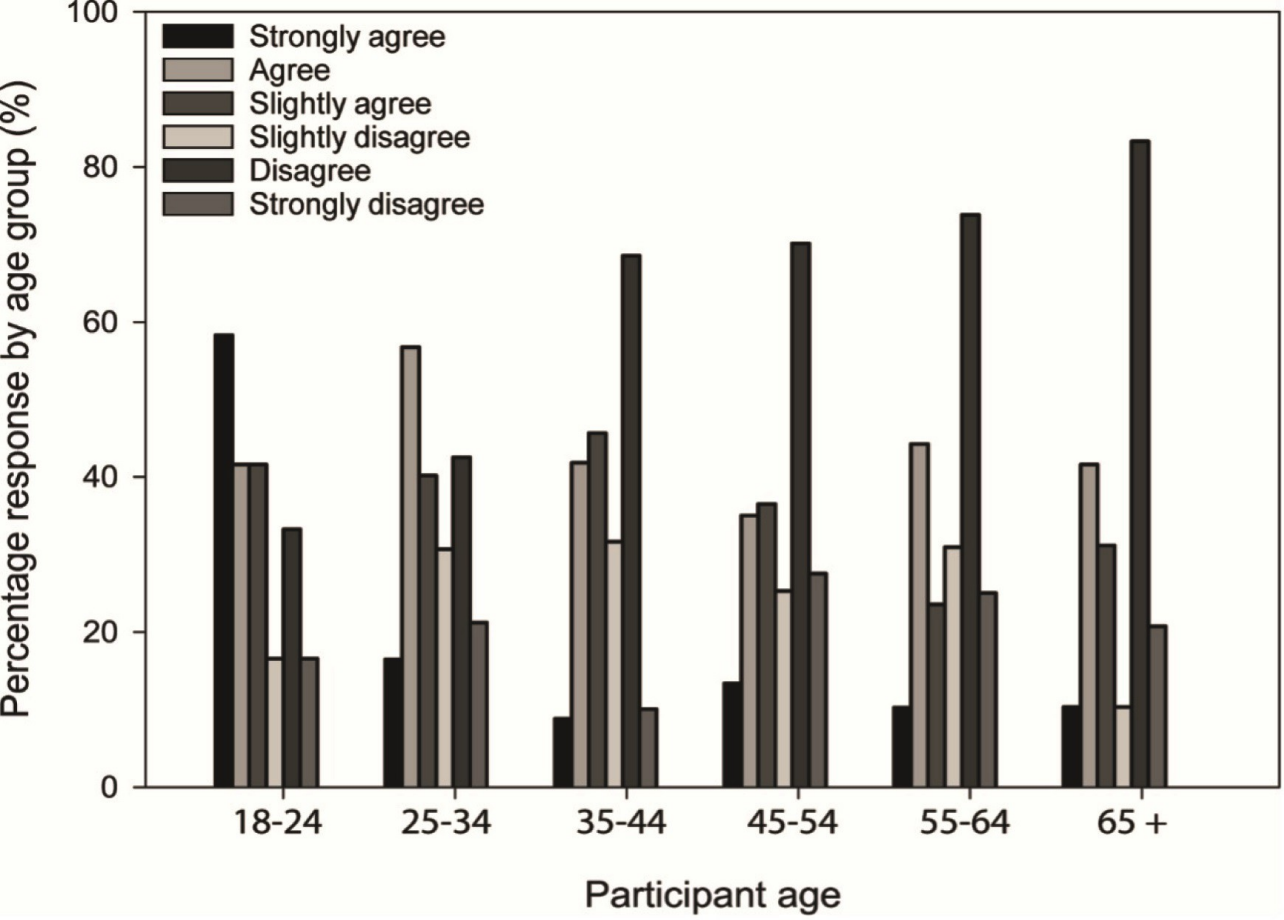
Tendency to cut short or cancel a swim due to water quality concerns according to age group.

Most participants (90%) recognised that water quality influences their overall swim experience. Most of these participants had not needed to cancel or cut short their open water swim due to poor water quality; however, this was a narrow majority over those who had cut short a swim due to water quality concerns (347 versus 301 participants). Overall, there was a significant, strong association between the likelihood of water quality influencing a participant’s swim experience and likelihood of cancelling a swim because of their concerns with water quality (P < 0.001, Φ_c_ = 0.15). Increased tendency to cut short or cancel a swim due to water quality concerns was not associated with any particular type of water environment (P > 0.05; Table 2) but was significantly associated with age, with younger swimmers, in particular 18–24 year olds, more likely to have cut short a swim (Fig 3; P < 0.01; Φ_c_ = 0.11).

**Table 2:** Responses to the question ‘There have been times when I’ve cut short or cancelled a wild swim because of concerns with water quality’ according to participants of different preferred water environments for open water swimming.

What is your preferred water environment for wild swimming?	There have been times when I’ve cut short or cancelled a wild swim because of concerns with water quality					
	**Strongly Agree %**	**Agree %**	**Slightly Agree %**	**Slightly Disagree %**	**Disagree %**	**Strongly Disagree %**
**Sea**	6.98	19.05	14.92	12.70	32.38	13.65
**Lakes/Lochs**	6.20	20.28	20.00	14.08	31.83	7.61
**Rivers**	6.82	25.00	18.18	13.64	27.27	9.09

Despite no significant association between preferred water environment and tendency to feel afraid when swimming, the majority of swimmers (38% of all participants) were in slight agreement that they sometimes felt afraid of the wider environment during their swim (55% tended to agree to an extent, whereas 44% tended to disagree to an extent; Table 3). Similarly, major risks to health, other than drowning, were not perceived to differ between water environments (P > 0.05), but there was a strong significant association with age, with 35–44-year-olds recognising potential wider risks to their health over and above any other age groups (P < 0.01; Φ_c_ = 0.15).

**Table 3:** Responses to the question ‘I sometimes feel afraid of the environment when wild swimming’ according to participants of different preferred water environments for open water swimming.

What is your preferred water environment for wild swimming?	I sometimes feel afraid of the environment when wild swimming					
	**Strongly Agree %**	**Agree %**	**Slightly Agree %**	**Slightly Disagree %**	**Disagree %**	**Strongly Disagree (%)**
**Sea**	3.49	15.56%	36.83%	11.75%	22.54%	9.84%
**Lakes/Lochs**	1.97	13.24%	39.72%	15.49%	23.38%	6.20%
**Rivers**	2.27	18.18%	36.36%	13.64%	20.45%	9.09%

**Fig 4:**
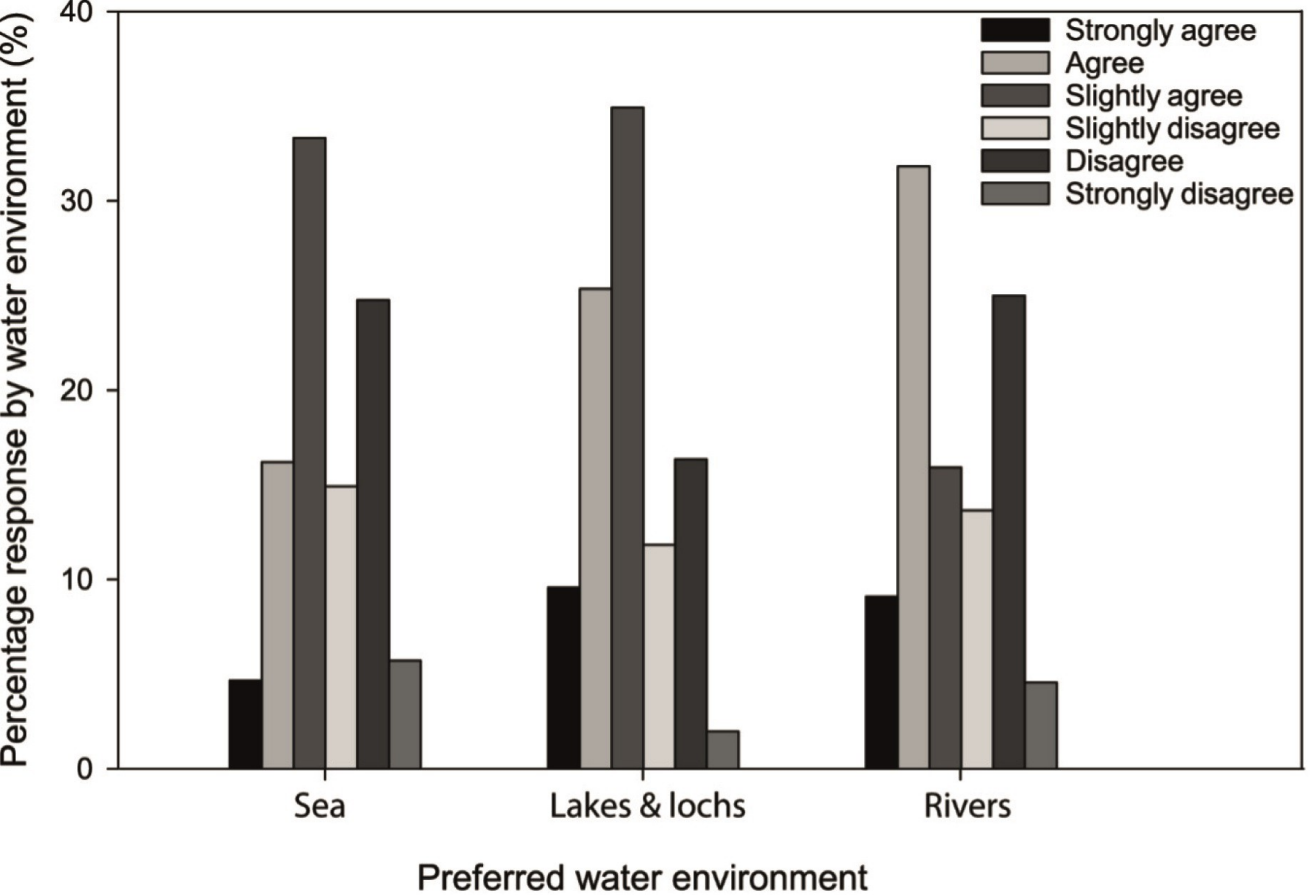
Concerned about possible environmental damage caused by increasing popularity of open water swimming? Level of agreement according to preferred water environment for swimming.

**Fig 5:**
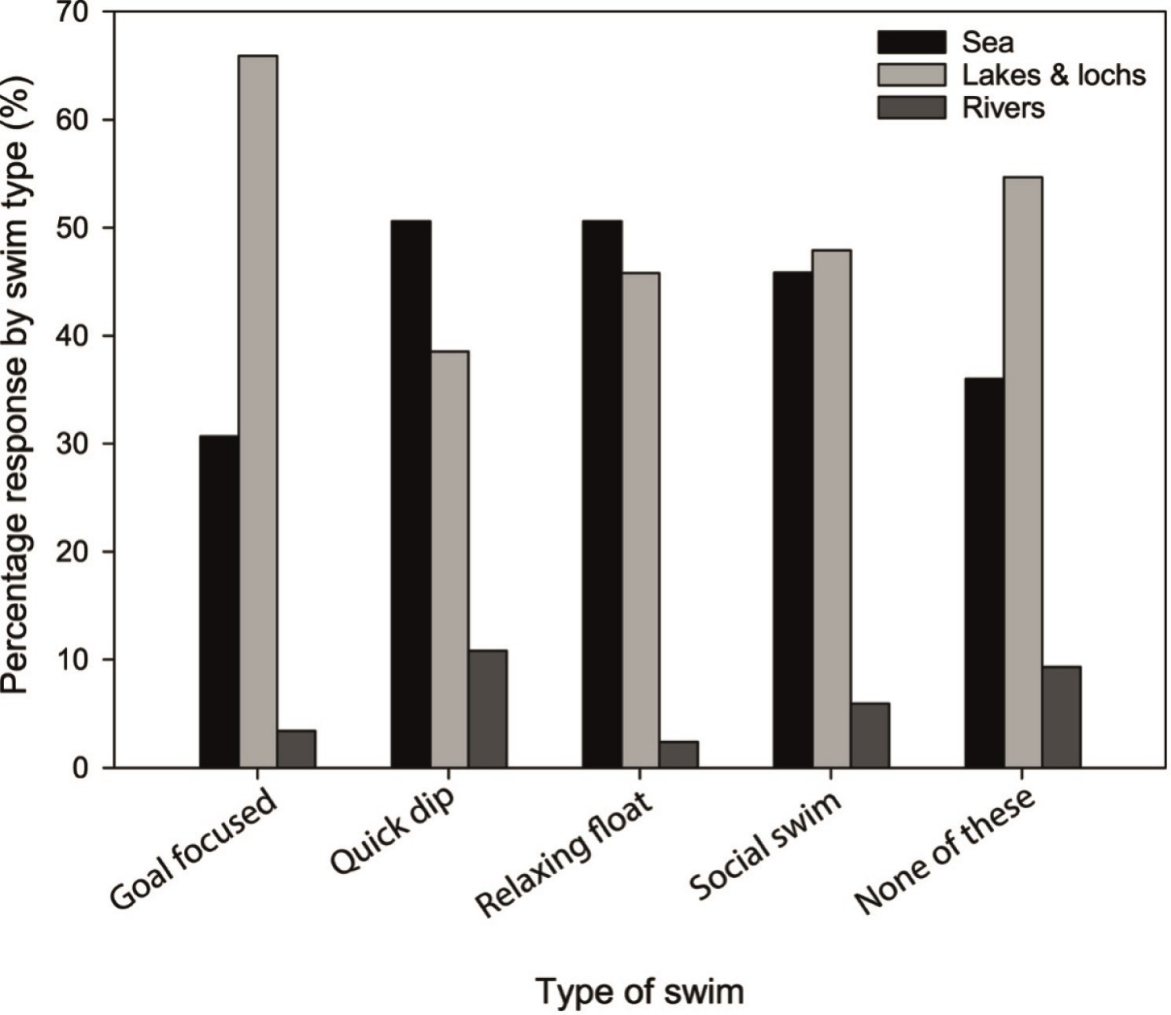
Preferred water environments associated with different typical swim styles.

Participants’ preferred type of water environment was significantly associated with their perceived level of concern regarding possible environmental damage caused by the increasing popularity of open water swimming (Fig 4). In general, those who preferred lake swimming reported greater concern compared to those participating in river or sea swimming (P < 0.001, Φ_c_ = 0.15). However, most participants perceived the risks to the environment from open water swimming to be minimal (73%) and there was no association between age group and perceptions of environmental risk (P > 0.05). The preferred water environment for open water swimming was also significantly associated with the typical swim undertaken (P < 0.001, Φ_c_ = 0.19; Fig 5). Most open water swimmers who regarded their typical swim as being goal-focussed preferred a lake environment (66%), with only 31% preferring the sea for this type of swim. The difference in preference for sea versus lakes was minimal for those who regarded their typical swim as being a social swim, 46% and 48%, respectively. For all categories of swim types, rivers were the least preferred environment, and they were more likely to be used for swims classified as a ‘quick dip’.

**Fig 6:**
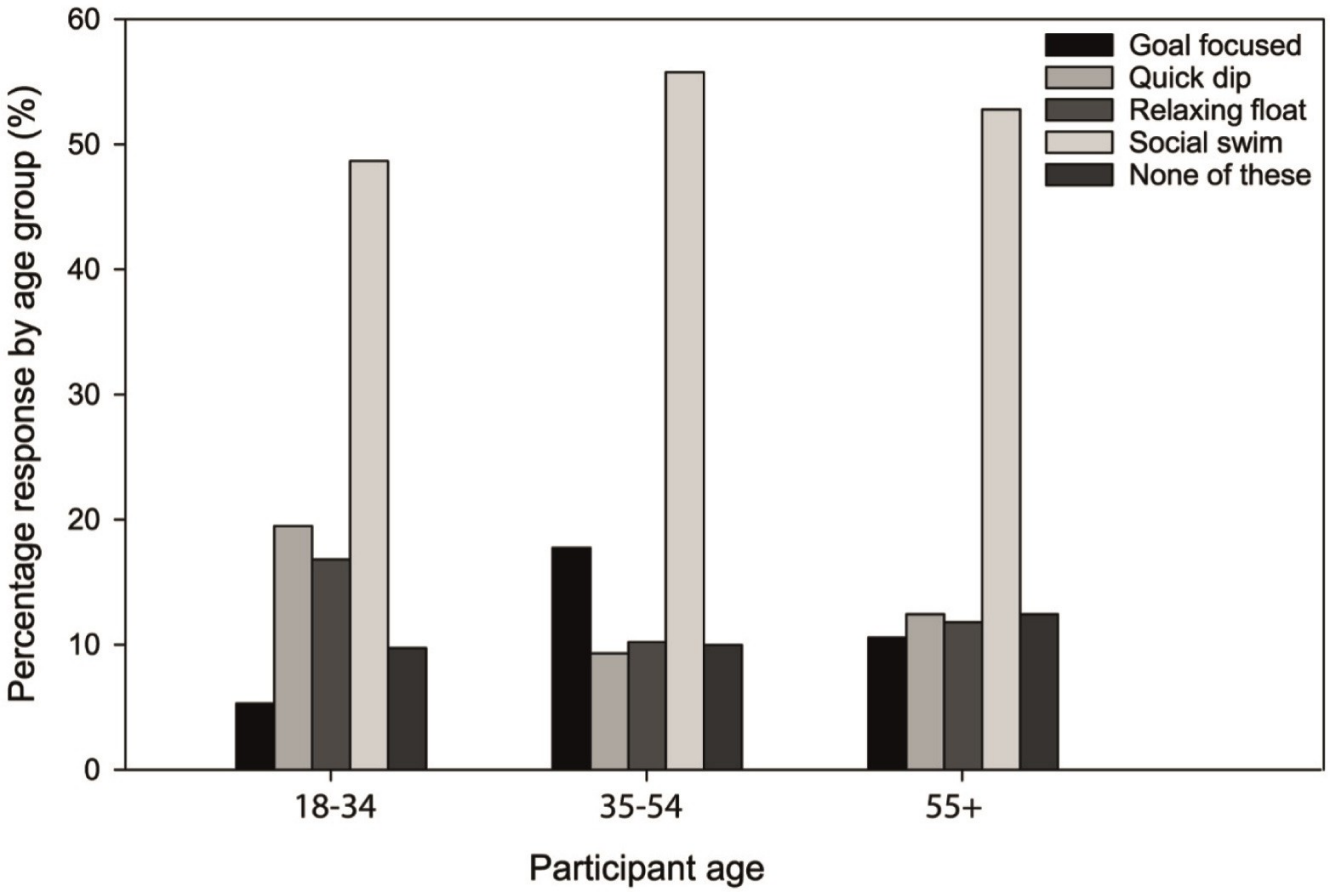
Distribution of typical swim styles according to age.

The most common categorisation of swim type across all responses was a social swim (54%). The proportion of responses to other swim types were much lower and evenly distributed: 12% for each of ‘relaxing float’, ‘goal-focused’ and ‘quick dip’, and 10% for none. The age of the participant was significantly associated with the type of swim (P < 0.01, Φ = 0.20). All age groups were dominated by participants who considered their activity to be a ‘social swim’, with ‘quick dips’ and ‘relaxing floats’ more likely in the 18–34 age group and goal-focused swims more likely in the 35–54 age group (Fig 6). The preferred water environment for open water swimming was significantly associated with the typical duration of the swim (P < 0.001, Φ_c_ = 0.15). In total, 67% of those who swam for more than one hour were likely to be lake swimmers, whereas shorter swims, of typical duration up to 30 minutes, were more likely to take place in the sea (53% of those who swam for 10–30 minutes and 52% of those who swam for less than 10 minutes). Irrespective of time spent open water swimming, rivers were the least popular environment for this recreational pursuit (Table 4).

**Table 4:** The preferred water environment associated with swims of different duration.

Duration of typical swim	Preferred water environment for open water swimming		
	Sea %	Lakes/Lochs %	Rivers %
More than 1 hours	31.7	67.07	1.22
>30 mins ‐ 1 hour	39.23	53.69	7.07
10 ‐ 30 mins	53.31	40.07	6.62
Less than 10 mins	52.38	45	7.76

In total, 86% of participants agreed that they assess possible risks to their health from the environment before swimming, and when reflecting on their last swim 89% of participants claimed they had a good understanding of the water quality at the swim location. Participants were asked to comment on how they judged the water quality of their wild swimming environment and the range of responses to this question were categorised into four key themes: (i) experience; (ii) social (media) groups; (iii) technological awareness; and (iv) no assessment. The most common approach was to rely on experience, with participants highlighting the importance of visual assessments of the water and recognising signs of algae, sewage or visible faecal matter. Others referred to timeframes, e.g., avoiding going swimming 24–72 hrs after rainfall because of increased likelihood of poor water quality. There was regular mention of using discussion groups (online or otherwise) to obtain information, allowing for an understanding of potential water quality prior to travel. This method allowed for more experienced swimmers to share their knowledge and experience with others. A theme of ‘technological awareness’ was also identified, with regular mention of online alerts or web tools. This included app-based tools used to highlight areas to avoid due to algal hotspots and online material available via the Scottish Environment Protection Agency (SEPA) reporting on water quality at designated bathing water sites, though frequently the responses mentioning the SEPA bathing water data commented that this would be used in conjunction with other sources of information.

## Discussion

Our study highlights important findings linking the demographics of open-water swimmers, the characteristics of their swim patterns and their perceptions of benefits and risks associated with this outdoor recreational pursuit which is growing in global popularity [25]. Improved understanding of benefits and risks of engaging with natural environments, such as blue spaces used for open water swimming, provides important evidence to help underpin the development of public health interventions and health-promoting policies [26].

The survey results provide a national overview of perspectives from across the Scottish wild-swimming community, albeit heavily skewed to female participant responses. While it is unlikely that the proportion of female participants responding to our survey is representative of the gender balance of the Scottish wild swimming community, the high participation rate of females relative to males is itself an interesting finding. Higher female representation is a recurrent theme in surveys of this nature; female response rates of 82% and 69% have been recorded in other recent surveys of open water swimmers [9, 12]. UK-wide profiling of the open water swimming community in 2020 reported 65% to be female [27]. We therefore report on patterns and viewpoints of the community surveyed and do not attempt to differentiate perceived benefits according to gender given the skew in participation.

Mental wellbeing benefits were perceived as more important than physical and social benefits across most age groups and across all swim types, reinforcing the value that participants associate with their connection with nature and perhaps reflecting the importance of a sense of place within the environment [28]. Open water swimming provides opportunities to promote mindfulness and restoration, further supporting the potential for this nature-based activity to improve mental wellbeing [10]. Evidence suggests a stronger association between physical activity and indicators of positive mental wellbeing when undertaken in natural environments relative to traditional built environment venues [29] and the use of natural environments links more strongly to stress reduction theory [30]. A stronger recognition of physical health benefits among older participants (> 65 years) likely reflects the greater importance they place on water as a favourable buoyant environment for exercise, reducing stress on knee and hip joints and making movement easier than physical activity on land [31]. Qualitative accounts suggest open water swimming has been shown to: support healthy ageing in elderly adults [32]; enable physical activity for older adults with physical limitations [11]; and ease physical menopausal symptoms [10]. While indoor swimming pools could facilitate this exercise, open water swimming offers other advantages, e.g., chlorine-free swimming, opportunities to engage with nature and health restoration potential for older swimmers [33]. For the majority of participants, across all age groups and all swimmer types, social interaction associated with open water swimming was rarely considered the most important benefit; however, the most common categorisation of swim style was termed a ‘social swim’, suggesting that while socialising was not the primary benefit for most, it remains a central element of swimming in natural environments [34].

Coastal waters and lakes (lochs) were identified as being the more popular blue space environments to support open water swimming; rivers were less frequently identified as being the preferred waterbody for this activity. The UK has over 600 designated bathing waters, which are sites identified as being popular for swimming and paddling [35]. The vast majority of these bathing waters are coastal, with a small number (< 20) located at inland freshwater lakes or lochs. In the UK there are currently only two designated river bathing waters, both located in England, which in part reflects the current low level of use of rivers for swimming in the UK. This contrasts with countries such as New Zealand, the USA and Canada, who adopt greater spatial sampling of microbial water quality across catchments, driven by cultural differences in river recreation [36].

River environments can provide important social spaces and opportunities for both water and waterside recreation, especially so under travel restrictions imposed by recent lockdowns [16]; however, in the UK they are principally managed for a range of other ecosystem services. From our study, it is unclear whether participants prefer coastal and lake environments over rivers due to waterbody physical characteristics influencing the swim experience or because of perceived risks specifically associated with river water quality and associated media interest (e.g., [37]). Common responses as to why participants preferred particular environments included convenience (e.g., proximity and access), water conditions (e.g., (dis)liking of salt water, calmer waters), safety (e.g., shallow depth, no currents, no jellyfish) and surrounding scenery. Detailed qualitative data derived from semi-structured interviews of open water swimmers has highlighted similar themes influencing preferred swimming environments [10]. While our study categorised participants by their preferred water environment for swimming, some responses highlighted a use of multiple types of blue space as opportunities arose.

Although most participants (i) suggested that water quality influences their overall swim experience; (ii) assessed risk before swimming; and (iii) claimed they had a good understanding of water quality issues (acknowledging advice to avoid swimming in the day(s) after heavy rainfall (see [38]), most swimmers considered risks to health (other than drowning) to be minimal. Therefore, open water swimmers may choose to prioritise wellbeing benefits over potential risks to physical health from water quality, as has been reported for surfers (c.f. [39]). However, sewage discharge into UK surface waters has recently received significant media attention with increased recognition of the frequency and magnitude of spills raising public awareness of the risks posed to water quality and downstream ecological and public health [40]. During 2020, in England alone, there were over 400,000 sewage discharges from 80% of combined sewer overflows monitored, totalling in excess of 3 million hours of discharge [41]. In 2022, over 14,000 sewage discharges were logged in Scotland [42]. Despite this, relative to convenience, water quality was less frequently mentioned as a factor influencing preferred water environments, reinforcing previous findings that water quality is a poor predictor of recreational hotspots, including for swimming [43]. This highlights scope to increase specific awareness among the open water swimming community of potential health risks from exposure to, and ingestion of contaminated water [10]. Younger swimmers were more likely to cancel swims when water quality was poor, suggesting a more critical and cautious approach to water quality and potential health risks among this age group [43], and potentially related to the growing presence of app-based technology capable of informing on historical and, in some cases, near real-time spatial and temporal water quality risks [39].

Those participants preferring to swim in lakes and lochs were more likely to do so for longer durations relative to sea and river swimmers and were more likely to be concerned about potential damage to the environment from the increasing popularity of this recreational activity. Spending more time in an environment can make an individual more aware of their surroundings and this may influence their perceptions of possible degradation of environmental quality [44]. Increased time in an environment would also facilitate greater contact with a larger number of people, which may potentially influence perceptions about increasing popularity of a particular location. The calmer setting of lakes and lochs, often cited in our survey as a reason for preferring these locations for swimming, may contribute to a perception of these environments being more vulnerable to disturbance from increased visitor numbers [45], whereas hydrologically dynamic environments such as flowing rivers and coastal tides may, in contrast, appear to be more resilient to increased usage because of that state of water flux.

The popularity of initiatives to promote public health via open water swimming or blue space engagement is growing [46]. Although our data and other risk-focused open water swimming studies [10, 12] suggest that risk awareness among open water swimmers in the UK is relatively high, illness and fatality related to immersion in open water remain a threat to public health [47]. Open water swimming and health research rarely captures the perspectives of new open water swimmers and often focuses on experienced swimmers, who are likely to be more risk-averse. This focus on experienced swimmers may explain the apparent ‘gap’ between risk awareness reported in qualitative studies and data on illness and fatalities linked to water environments. Policymakers and practitioners should be aware of this research gap when developing policy (e.g., social prescribing programmes) and assessing quality assurance and liabilities of blue-care activities [22]. Further research aiming to understand the risks of informal or inexperienced open water swimming would add substantial value to the current evidence base.

Our research provides cross-sectional evidence from a survey of Scottish open water swimmers. The large sample size provides rare insight into how this community perceives benefits and risks of open water swimming to both public health and the environment. However, our study was also subject to limitations, which in turn offer opportunities for future investigation. The high proportion of female participants and the high mean household income relative to the Scottish population may limit the generalisability of our findings. The cross-sectional design, deployed in 2021, captured participant data during the COVID-19 pandemic; understanding whether perceived benefits and risks vary over time, e.g. post pandemic, would offer further insight as to how representative the timing of our survey was and provide opportunities to better understand causality. Furthermore, the data collection period occurred in the summer over a month-long period and it would be interesting to deploy the survey through the seasons to determine whether during different temperature, daylight and weather regimes participant recall of their most recent swim influenced perceptions of benefits and risks of open water swimming.

## Conclusion

Our study contributes to the growing evidence that open water swimming can uniquely benefit mental and physical wellbeing, but also provides key insight into how risks to public health and the environment resulting from this recreational pursuit are perceived. This is important to help design and develop scalable nature-based health interventions, including wellness services via social prescribing programmes, that fully account for potential trade-offs in blue care. However, we currently lack a detailed understanding of how perceived trade-offs in benefits versus risks associated with open water swimming vary in space and time. While challenging, longitudinal analysis of the open water swimmer community would be advantageous to understand how perceptions of risks and benefits vary over a swimmer’s life-course and how spatial and temporal changes in water quality driven by, e.g., climate change and government policy, influence those perceptions.

## Supporting information

S1 File A copy of the online survey questions. (PDF)
