# Supplementary material for: Self-reported benefits and risks of open water swimming to health, wellbeing and the environment: Cross-sectional evidence from a survey of Scottish swimmers
Source: PLoS One. 2023 Aug 28;18(8):e0290834. doi: 10.1371/journal.pone.0290834 (PMC10461842; doi:10.1371/journal.pone.0290834)
Supplement: S1 File — (PDF) [file pone.0290834.s001.pdf]

Please don't select more than 1 answer(s) per row.

Please select at least 2 answer(s).

	Please tick the boxes to confirm
I agree to take part in the study	☐
I am 18 yrs old or over	☐

Page 2: Personal information

2. How old are you?

- 18-24
- 25-34
- 35-44
- 45-54
- 55-64
- 65-74
- 75 or older

3. What is your gender?

- Female
- Male
- Other
- Prefer not to say

4. In what region of Scotland do you live? ✱ *Required*

5. What is your approximate annual household income?

- Less than £20k
- £20-29,999
- £30-39,999
- £40-49,999

- £50-59,999
- £60-69,999
- Over £70k
- Prefer not to say

Page 3: Wild swimming background

6. How long have you been wild swimming? (please state approx length of time in years / months)

7. On average, what distance do you travel to go wild swimming?

- Up to 1 km
- More than 1 km but less than 5 km
- More than 5 km but less than 10 km
- More than 10 km but less than 20 km
- More than 20 km

8. What is your preferred water environment for wild swimming?

- Sea
- Lochs and lakes
- Rivers

9. Regarding your answer to the previous question, why is that?

10. Over the last 12 months, approximately what proportion of your wild swimming time have

you spent in each of these environments? (answer should total 100%)

Sea	
Loch/lake	
River	

11. On a typical swim, how much time do you spend in the water?

- More than 2 hours
- between 1-2 hours
- 30 mins - 1 hour
- 10 - 30 mins
- Less than 10 mins

12. And on average, how often do you go wild swimming?

- Daily
- Few times a week
- Once a week
- Few times a month
- Once every couple of months

13. Which of the following would best describe your typical swim?

- Goal focused distance swim
- Quick dip
- Relaxing float
- Social swim
- None of these

Page 4: Swim experiences and associated perceptions

14. Which of the following benefits do you gain from your wild swimming activities (tick all that apply)

- ☐ Physical benefits
- ☐ Mental wellbeing benefits
- ☐ Social benefits

15. Which of the following is the most important benefit you gain from wild swimming?

- ○ Social interaction
- ○ Physical health benefits
- ○ Mental wellbeing benefits

16. How strongly do you agree/disagree with each of the following statements:

Please don't select more than 1 answer(s) per row.

Please select at least 3 answer(s).

	Strongly Agree	Agree	Slightly Agree	Slightly Disagree	Disagree	Strongly Disagree
The social benefits of wild swimming are more important to me than the physical health benefits	☐	☐	☐	☐	☐	☐
The social benefits of wild swimming are more important to me than the mental wellbeing benefits	☐	☐	☐	☐	☐	☐

The mental wellbeing benefits of wild swimming are more important to me than the physical health benefits	☐	☐	☐	☐	☐	☐

17. How strongly do you agree/disagree with each of the following statements:

Please don't select more than 1 answer(s) per row.

Please select at least 3 answer(s).

	Strongly Agree	Agree	Slightly Agree	Slightly Disagree	Disagree	Strongly Disagree
I am concerned about possible environmental damage caused by increasing popularity of wild swimming	☐	☐	☐	☐	☐	☐
There have been times when I've cut short or cancelled a wild swim because of concerns with water quality	☐	☐	☐	☐	☐	☐
I had to adapt my wild swimming routine because of COVID-19	☐	☐	☐	☐	☐	☐

18. If you had to adapt your wild swimming routine because of COVID-19 can you describe how? If you did not have to adapt, do you want to comment?

19. How strongly do you agree/disagree with each of the following statements:

Please don't select more than 1 answer(s) per row.

Please select at least 3 answer(s).

	Strongly Agree	Agree	Slightly Agree	Slightly Disagree	Disagree	Strongly Disagree
I sometimes feel afraid of the environment when wild swimming	☐	☐	☐	☐	☐	☐
Other than drowning, wild swimming does not present any major risk to my health or safety	☐	☐	☐	☐	☐	☐
The risks to the environment from wild swimming are minimal	☐	☐	☐	☐	☐	☐

Page 5: About your last swim.....

20. Thinking back to your last swim, approximately how long were you in the water (hrs, mins)?

21. Thinking back to your last swim, did you swim alone?

22. Thinking back to your last wild swim.......

Please don't select more than 1 answer(s) per row.

Please select at least 7 answer(s).

	Strongly Agree	Agree	Slightly Agree	Slightly Disagree	Disagree	Strongly Disagree
You found it beneficial for your physical wellbeing	☐	☐	☐	☐	☐	☐
You found it beneficial for your mental wellbeing	☐	☐	☐	☐	☐	☐
You found it beneficial for your social wellbeing	☐	☐	☐	☐	☐	☐
The quality of the water played an important role in your overall swimming experience	☐	☐	☐	☐	☐	☐

You had a good understanding of the water quality where you were swimming	☐	☐	☐	☐	☐	☐
You assessed possible risks to your health from the environment before swimming	☐	☐	☐	☐	☐	☐
You considered possible risks to the environment from your swim before entering the water	☐	☐	☐	☐	☐	☐

23. What information do you use to understand the water quality of your wild swimming environment, if any?

Page 6: Final page

Thank you very much taking the time to complete the survey. Your participation is greatly appreciated. For any further questions, please contact c.w.mcdougall@stir.ac.uk.

Key for selection options

4 - In what region of Scotland do you live?

- Inverclyde
- Renfrewshire
- West Dunbartonshire
- East Dunbartonshire
- City of Glasgow
- East Renfrewshire
- North Lanarkshire
- Falkirk
- West Lothian
- City of Edinburgh
- Midlothian
- East Lothian
- Clackmannanshire
- Fife
- City of Dundee
- Angus
- Aberdeenshire
- City of Aberdeen
- Moray
- Highland
- Western Isles
- Argyll & Bute
- Perth & Kinross
- Stirling
- North Ayrshire
- East Ayrshire
- South Ayrshire
- Dumfries & Galloway
- South Lanarkshire
- Scottish Borders
- Orkney Islands
- Shetland Islands

21 - Thinking back to your last swim, did you swim alone?

Yes

No
